# Neighborhood effects on dietary behaviors—evidence from older adults in China

**DOI:** 10.3389/fnut.2022.974471

**Published:** 2022-09-28

**Authors:** Chang Liu, Hao Yu

**Affiliations:** College of Economics and Managerment, Nanjing Forestry University, Nanjing, China

**Keywords:** dietary behaviors, China, neighborhood effects, nutrient intake, older adults

## Abstract

Individual neighborhood environment is an important predictor of dietary behavior. Using data from four waves of the China Health and Nutrition Survey (CHNS, 2004–2011), this study applied a panel data approach to examine the effects of neighborhood diet quality on the eating behaviors of older adults living in the same community. Results of the fixed effects estimation indicated a significant neighborhood effect within the community, and neighborhoods with high-quality diets had a significantly positive effect on the eating behavior of the elderly. The neighborhood effects on elderly eating behaviors were manifested in improved dietary structure, including decreased consumption of cereals and increased consumption of vegetables and fruits, as well as meat, eggs, and dairy products. In terms of nutrient intake, there was a significant increase in protein intake, and hence, a greater percentage of calories from protein. The estimation results were robust when different estimation methods or diet quality measures were used. Future policies for improving diet quality should consider neighborhood-level conditions, especially in rural areas where residents are closely connected and socially interact with one another.

## Introduction

Population aging has become a public health issue in China. According to the National Bureau of Statistics of China, the number of people aged 65 and over is 200.56 million, accounting for approximately 14.19% of the total population of the mainland in 2021. The development of chronic diseases in the elderly population is closely related to dietary structure and health behaviors. A low-quality diet and lack of physical activity are major risk factors for overweight/obesity, diabetes, hypertension, cardiovascular diseases, and other health-related consequences (1–4). Dietary patterns have become a key topic in health research, and many studies have focused on the association between dietary intake and obesity (5–8). For example, Papandreou et al. (7) found that the intake of energy, protein, carbohydrates, and thiamine was positively associated with obesity in children, whereas children with dietary iron deficiency were more likely to be obese after adjusting for energy intake. Through a cross-sectional household-based study, Zou et al. (8) extracted five dietary patterns, including “cereal, animal, and plant foods,” “high-protein foods,” “plant foods,” “poultry” and “beverages” dietary patterns among Chinese adults and found that the “cereal, animal, and plant foods” and “beverages” dietary patterns increased the intake of protein and fat and the intake of carbohydrates, which ultimately lead to obesity. Moreover, dietary patterns have been shown to be associated with the occurrence of non-communicable diseases [(9–11)]. For instance, the GBD 2017 Diet Collaborators (12) conducted a systematic study and found that high sodium intake and low whole grain and fruit intake were major dietary risk factors for death and disability-adjusted life expectancy in many countries worldwide. Similarly, Wang et al. (9) identified five dietary patterns and found that “protein dietary pattern,” “balanced dietary pattern,” and “beans dietary pattern” show protective effects on cardio-cerebrovascular disease, whereas “prudent dietary pattern” and “traditional dietary pattern” were positively associated with hypertension.

In terms of factors influencing dietary behaviors, the residential environment is an important predictor, in addition to individual and household characteristics (13–15). Most existing research has focused on home and neighborhood built environments to differentiate between dietary behaviors among adolescents and adults (16–19). For instance, Ho et al. (17) found that fast food shops, restaurants, and convenience stores that are available to adolescents in the neighborhood, especially poor ones, may have a negative effect on their dietary intake. Similarly, Berge et al. (20) found that healthy neighborhoods and home environments were associated with adolescents' healthy dietary intake and low BMI. Additionally, neighborhood socioeconomic conditions and external shocks may lead to changes in individuals' dietary patterns (21). Mayne et al. (22) focused on the associations of neighborhood social environment exposures, including perceived safety, collective efficacy, and crime, with dietary intake and found that high perceptions of the neighborhood environment were associated with high consumption of some healthy foods among preschool-aged children and their mothers.

To better understand the pathway to healthy aging, not only the neighborhood built environment but also the dietary behaviors of neighbors should be considered, especially in areas where residents are closely connected and socially interact with one another. However, few studies have addressed the relationship between dietary behavior and neighbors. Except for Leonard et al. (18), their research found that neighbors' dietary patterns are related to individuals' food consumption (higher fruit and vegetable intake) while controlling for food sources and neighborhood built environment.

This study investigates whether neighborhood diet quality is a predictor of dietary behaviors in older life stages in the context of China. Using data from the China Health and Nutrition Survey (CHNS, 2004–2011), we applied a fixed effects approach to account for unobserved individual heterogeneity. We found significant and positive neighborhood effects on dietary behavior among older adults. Neighborhood diet quality had a significant positive effect on the eating behavior of the elderly. This study provides evidence for a consistent association between neighborhood effects and dietary behaviors by dividing the sample into subgroups. However, no significant gender or regional differences were observed in the heterogeneity analysis.

The remainder of this paper is organized as follows. Section Materials and methods describes the empirical framework, data, and measurements of the study variables. Section Results presents our estimation results. The final section concludes the paper and discusses future policies.

## Materials and methods

### Data source

The data employed in this study were derived from the China Health and Nutrition Survey (CHNS) conducted by the Carolina Population Center at the University of North Carolina at Chapel Hill and the National Institute for Nutrition and Health at the Chinese Center for Disease Control and Prevention. The CHNS is a longitudinal survey covering detailed information, such as background demographics, work activities and income, health service and disease history, and food consumption. A multistage random cluster method was used to draw samples from nine provinces of China, including Henan, Hubei, Heilongjiang, Liaoning, Shandong, Guizhou, Jiangsu, Guangxi, and Hunan. The first round of CHNS was collected in 1989. Nine additional panels were collected for 1991, 1993, 1997, 2000, 2004, 2006, 2009, 2011, and 2015.

**Figure 1:**
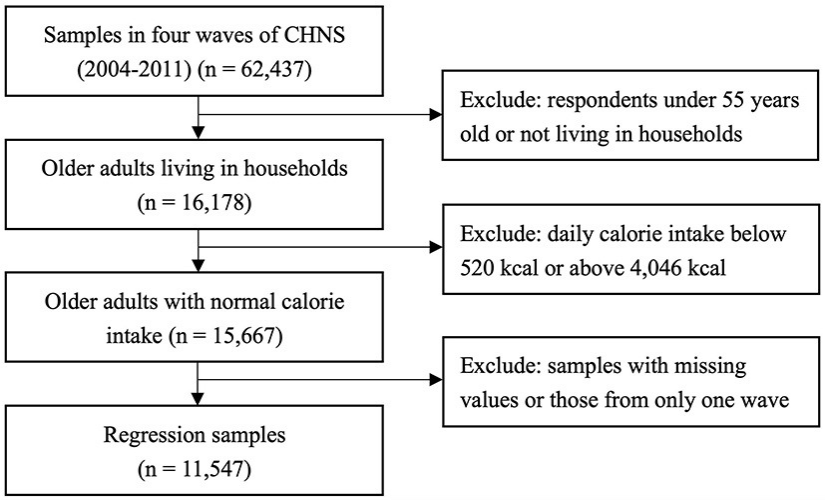
Flow chart of study population selection.

Notably, the CHNS adopted a new food code system after the wave 2004, which is consistent with the coding in the Chinese Food Composition. Hence, four waves of data from 2004, 2006, 2009, and 2011 were used to construct the panel dataset^1^. In this study, we focused on adults aged 55 years or older in urban and rural areas. After excluding samples with missing values and outliers, 11,547 observations were used. Specifically, the sample sizes in 2004 were 2,264, those 2006, 2009, and 2011 in 2,817, 3,387, and 3,079, respectively. The detailed sampling process is illustrated in Figure 1.

### Measures

#### Dietary behaviors

Based on the China Food Composition (23, 24), and detailed food consumption records in the CHNS, we constructed several indicators to describe the dietary behaviors of older adults.

##### Diet quality

In this study, Chinese Healthy Eating Index (CHEI) was used to describe overall diet quality for Chinese people, which is developed on the basis of the Healthy Eating Index (HEI)^2^ (25, 26). According to the CHEI, diet quality is assessed from daily food consumption, which can be divided into adequacy components (12 food groups) and limitation components (5 groups) (see Table 1). Recommended amounts were converted into standard portions per 1,000 calories (SP/1,000 kcal) at different caloric levels^3^ for each adequacy or limitation component. Scoring for CHEI components is based on energy density, among which cooking oils, sodium, fruits, and dairy are assigned 10 points^4^, and other components are assigned 5 points. The sum of an older individual's CHEI score ranges from 0 to 100 points, with higher scores indicating higher food quality. Details of the scoring are described in Yuan et al. (25).

**Table 1:** Components of diet quality indicators.

**Diet quality indicators**	**Components**
Chinese Healthy Eating Index (CHEI)	***Adequacy components***
	Total grains; Whole grains and mixed beans; Tubers; Total vegetables; Dark vegetables; Fruits; Dairy; Soybeans; Fish and seafood; Poultry; Eggs; Seeds and nuts
	***Limitation components***
	Red meat; Cooking oils; Sodium; Added sugars; Alcohol
Chinese Diet Quality Distance (DQD)	Cereals; Vegetables and fruits; Dairy products, soybean, and soybean products; Animal food; Condiments and alcoholic beverages; Dietary variety*; Drinking water

*The dietary variety of DQD was based on 12 food subgroups, and −1/0 points were recorded for each subgroup according to daily intake.

In addition to CHEI, the Chinese Diet Quality Distance (DQD) was constructed with reference to the Chinese Dietary Balance Index-07 (DBI-07) (28, 29). Similarly, DQD contains seven food groups (Table 1). For each food group, if a respondent's daily intake reached the recommended intake level, a score of 0 points was recorded. Negative (ranging from −12 to −1) and positive scores (ranging from 1 to 12) were recorded to evaluate inadequate and excessive food intake. Details of the scoring are described in Xu et al. (29). DQD was calculated by summing the absolute values of the positive and negative scores of each DBI-07 component, ranging from 0 to 84 points. Contrary to CHEI, higher points of DQD indicate a less balanced diet.

##### Food consumption

In addition to diet quality, we computed the daily food consumption of each participant. The Chinese Food Guide Pagoda (2016) follows the principle of a balanced diet and reflects a nutritionally desirable basic food composition. Based on the structure of the Chinese Food Guide Pagoda (2016), we divided daily food consumption into three food groups: (1) cereals (and cereal products); (2) vegetables and fruits; and (3) meat, eggs, and dairy products. Therefore, the dietary behavior of older adults can be evaluated by observing their daily intake of different food groups.

##### Nutrient intake

Furthermore, we computed the daily intake of four macronutrients for each older adult: (1) dietary energy, (2) carbohydrate, (3) fat, and (4) protein, among which dietary energy^5^ is an overall indicator that measures an older individual's daily energy intake. Carbohydrates, fats, and proteins are the three main macronutrients that an individual needs to maintain their body's structure and systems. Moreover, the distribution of total calories, expressed as a percentage of calories obtained from carbohydrates, fats, and proteins, was used to assess the nutrient structure.

#### Neighborhood dietary behaviors

In this study, we defined the neighborhood effect as the average diet quality of all adults living in the same community, measured by CHEI and DQD. As mentioned above, both indicators depict the overall evaluation of an individual's eating behavior. Hence, a higher average CHEI value represents better dietary behaviors in the neighborhood, whereas a higher DQD value describes worse dietary behaviors in the neighborhood.

#### Control variables

To investigate neighborhood effects on dietary behaviors among older adults, we controlled for the following variables. (1) Individual characteristics: age, marital status (married/others), education level (in years), working status (yes/no), and number of chronic diseases, which is a proxy for the health endowment of each older adult. (2) Household characteristics: the number of household members, per capita household income (in thousands of yuan), age, gender (male/female), and education level (in years) of the main cook in the household. (3) Community-level characteristics: the number of grocery stores and supermarkets, and the type of road within communities (dirt/stone, gravel, or mixed material/paved road). Following Liu et al. (15), we included year-month dummy variables to eliminate potential seasonal variations in food consumption. In addition, we included interactive fixed effects of each city and survey time to reduce the potential bias caused by time-varying unobservables at the city level, such as external shocks or regional policies.

### Method

To estimate the effects of neighborhood diet quality on the dietary behaviors of elderly people in China, we specified a high-dimensional fixed effects model, which can be written as follows:


(1) $$\begin{array}{l} Dietary_{it}\;=\;\beta_{0}\;+\;\beta_{1}MDietary_{jt}\;+\;\beta_{2}X_{it}^{I}\;+\;\beta_{3}X_{it}^{H}\;+\;\beta_{3}X_{it}^{V}\\ \;+\;u_{i}\;+\;T_{t}\;+\;\delta_{c}w_{t}\;+\;\varepsilon_{it} \end{array}$$

where *Dietary*_*it*_ represents individual *i*'s dietary behavior in year *t*, including both diet quality and dietary intake of his/her meals, and *MDietary*_*jt*_ represents neighborhood effects, measured by the average value of the dietary indicator for all adults living in the same community in year *t*. Other control variables included individual characteristics $X_{it}^{I}$, household characteristics $X_{it}^{H}$, and community-level characteristics $X_{it}^{V}$. *u*_*i*_ denotes the individual fixed effects; *T*_*t*_ represents time fixed effects, including year-month dummies; δ_*c*_*w*_*t*_ is interactive fixed effects, namely, an interactive term of city dummies and year dummies; and ε_*it*_ is the idiosyncratic error term.

In particular, the current setting of the fixed effects model controls for time-invariant unobservables, such as dietary habits and food preferences, through within-group differencing, which removes omitted variable bias. Moreover, time and interactive fixed effects are included in the regressions, accounting for potential seasonal variations in food consumption and time-varying unobservables at the city level, such as external shocks and regional policies, respectively. Considering the potential correlation in residuals across individuals living in the same community, we reported robust standard errors by clustering at the community level. Singletons were excluded from the regression analysis to avoid incorrect inferences (31, 32). In addition, given the imperfection of fixed effects models for potential endogeneity problems, we attempt to construct instrumental variables and apply two-stage least squares (2SLS) to estimate the neighborhood effects in the robustness check.

## Results

### Descriptive analysis

Table 2 shows descriptive statistics of the variables, among which older adults are grouped into two sub-samples: (1) urban older adult and (2) rural older adults. The average age of older adults in the sample was about 66 years old, among which around 47% are male and 53% were female. Compared with the rural elderly, the urban elderly are about 1 year older and have better dietary behaviors. Specifically, they had higher quality diet, measured by CHEI and DQD indicators, and better dietary structure, namely, lower consumption of cereals and higher consumption of vegetables and fruits as well as meat, eggs, and dairy products. Hence, urban adults have higher nutrient intake of fat and protein but less intake of carbohydrates. In terms of dietary energy, the rural elderly had a slightly higher daily intake than their urban counterparts. In general, urban elderly belong to higher socioeconomic groups, with more years of education and higher per capita household income. However, in terms of health endowment, urban elderly suffer from more chronic diseases than rural elderly. Urban–rural differences are also evident in terms of community environments. The percentage of having paved road is higher in urban (88%) than in rural areas (75%).

**Table 2:** Summary statistics.

**Variables**	**Unit**	**All**		**Urban**		**Rural**	
		**Mean**	**S.D**.	**Mean**	**S.D**.	**Mean**	**S.D**.
**Dietary behaviors**							
CHEI	point	43.59	10.12	47.33	10.85	41.60	9.11
DQD	point	42.24	9.14	38.47	8.99	44.25	8.57
Cereals	g	417.31	188.01	376.95	168.69	438.75	194.13
Vegetables and fruits	g	337.98	184.38	356.13	194.78	328.34	177.87
Meat, Eggs and Dairy products	g	130.13	113.23	177.02	130.86	105.22	93.54
Dietary energy	kcal	2001.64	630.97	1940.82	611.35	2033.95	638.84
Carbohydrate	g	279.37	104.04	248.28	92.12	295.88	106.22
Fat	g	68.24	34.84	74.91	36.37	64.70	33.46
Protein	g	61.24	22.50	63.45	23.79	60.06	21.69
Share of calorie obtained from carbohydrate	%	56.17	11.86	51.62	11.58	58.58	11.29
Share of calorie obtained from fat	%	30.47	11.42	34.34	11.31	28.41	10.93
Share of calorie obtained from protein	%	12.37	2.89	13.21	3.21	11.92	2.60
**Individual characteristics**							
Age	year	65.63	7.85	66.13	8.13	65.37	7.68
Male	1 = male, 0 = female	0.47	0.50	0.47	0.50	0.47	0.50
Married	1 = married, 0 = others	0.81	0.39	0.82	0.39	0.80	0.40
Education in years	year	5.24	4.41	6.65	4.88	4.48	3.93
Dummy variable for work	1 = work, 0 = others	0.35	0.48	0.17	0.37	0.44	0.50
Number of chronic diseases	number	0.34	0.60	0.45	0.68	0.28	0.55
**Household characteristics**							
Number of household members	number	3.37	1.82	3.03	1.50	3.55	1.95
Per capita income	thousand yuan	11.50	13.02	15.98	13.35	9.12	12.20
Age of household cook	year	61.61	10.80	62.13	10.48	61.34	10.95
Male household cook	1 = male, 0 = female	0.20	0.40	0.24	0.43	0.18	0.38
Household cook's education in years	year	5.11	4.36	6.71	4.68	4.26	3.93
**Community-level characteristics**							
Number of grocery stores and supermarkets	number	17.73	28.36	17.80	25.63	17.69	29.71
Characteristics of the road 1	1 = dirt, 0 = others	0.04	0.20	0.03	0.17	0.05	0.21
Characteristics of the road 2	1 = stone, gravel, or mixed material, 0 = others	0.16	0.37	0.10	0.29	0.20	0.40
Characteristics of the road 3	1 = paved road, 0 = others	0.80	0.40	0.88	0.33	0.75	0.43

### Neighborhood effects and dietary behaviors of older adults

Table 3 presents the fixed effects estimates. As shown in column (1), the coefficient of neighborhood CHEI is positive and statistically significant, which indicates healthier diets among older individuals who live in a community of neighborhoods with a high-quality diet. Columns (2)–(4) in Table 3 show that the neighborhood's diet quality is associated with older adults' improved food structure. Specifically, older adults have decreased consumption of cereals and increased consumption of vegetables and fruits, as well as meat, eggs, and dairy products.

**Table 3:** Estimation of neighborhood effects on dietary behaviors of older adults (fixed effects estimators).

**Variables**	**Diet quality**	**Food consumption**		
	**CHEI**	**Cereals**	**Vegetables and fruits**	**Meat, eggs and dairy products**
	**(1)**	**(2)**	**(3)**	**(4)**
Neighborhood CHEI	0.633*** (0.047)	−0.089 (1.077)	2.563** (1.202)	1.797*** (0.497)
Square of age	−0.337 (0.259)	−10.375** (5.026)	−2.959 (5.377)	1.569 (3.275)
Married	1.256** (0.559)	15.733* (9.018)	−11.095 (10.211)	−7.430 (6.294)
Education in years	0.014 (0.056)	−1.588 (1.266)	3.522*** (1.214)	1.371** (0.605)
Dummy variable for work	0.759*** (0.289)	11.721* (6.705)	4.974 (5.989)	3.240 (3.262)
Number of chronic diseases	−0.147 (0.217)	−1.223 (3.987)	−9.597** (4.444)	0.326 (2.291)
Number of household members	0.576*** (0.108)	0.092 (2.440)	4.459** (2.222)	0.305 (1.211)
Per capita income	0.028** (0.011)	−0.429** (0.194)	0.656** (0.277)	0.329*** (0.122)
Age of household cook	−0.018 (0.015)	−0.312 (0.342)	−0.201 (0.323)	0.158 (0.168)
Male household cook	−0.401 (0.353)	0.368 (8.055)	−18.954*** (7.209)	2.410 (4.301)
Household cook's education in years	0.032 (0.060)	−0.649 (1.274)	−0.821 (1.266)	0.504 (0.752)
Number of grocery stores and supermarkets	0.006 (0.006)	0.277* (0.155)	0.133 (0.109)	0.056 (0.061)
Characteristics of the road 1	−1.388*** (0.505)	12.040 (15.701)	−4.766 (11.670)	−2.632 (6.485)
Characteristics of the road 2	−0.418 (0.376)	−4.556 (9.318)	−7.627 (9.335)	−7.889* (4.478)
Constant	27.493** (11.554)	888.987*** (216.908)	341.458 (244.950)	−38.462 (145.341)
Individual FE	Yes	Yes	Yes	Yes
Time FE	Yes	Yes	Yes	Yes
Interactive FE	Yes	Yes	Yes	Yes
Observations	11,547	11,547	11,547	11,547

***p < 0.01, **p < 0.05, *p < 0.1. Standard errors in parentheses are clustered at the community level. Interactive fixed effects are interactive terms for city fixed effects and year fixed effects.

In addition to neighborhood effects, individual socioeconomic status is a significant predictor of dietary behavior. Older adults with higher educational levels tend to consume more vegetables and fruits, as well as meat, eggs, and dairy products. Higher household income is associated with improved diet quality among older adults. Elderly people consume more vegetables and fruits, as well as meat, eggs, and dairy products, but consume fewer cereals, indicating an improved dietary structure. The variables that measured access to community food were statistically significant. Communities with more grocery stores and supermarkets are related to improved diet quality and food consumption among older residents, which is consistent with the literature on the influence of community context on dietary behaviors (16–18).

### Neighborhood effects and nutrient intake of older adults

We further estimated the fixed effects models with each older adult's nutrient intake as the dependent variable. To save space, only the neighborhood CHEI was retained, and the estimation results are presented in Table 4.

**Table 4:** Estimation of neighborhood effects on nutrient intakes of older adults (fixed effects estimators).

**Variables**	**Nutrients intake**				**Share of calories obtained from**		
	**Dietary energy**	**Carbohydrate**	**Fat**	**Protein**	**Carbohydrate**	**Fat**	**Protein**
	**(1)**	**(2)**	**(3)**	**(4)**	**(5)**	**(6)**	**(7)**
Neighborhood CHEI	−0.212 (4.043)	0.074 (0.652)	−0.348* (0.191)	0.435*** (0.142)	−0.017 (0.060)	−0.097 (0.060)	0.084*** (0.014)
Control variables	Yes	Yes	Yes	Yes	Yes	Yes	Yes
Individual FE	Yes	Yes	Yes	Yes	Yes	Yes	Yes
Time FE	Yes	Yes	Yes	Yes	Yes	Yes	Yes
Interactive FE	Yes	Yes	Yes	Yes	Yes	Yes	Yes
Observations	11,547	11,547	11,547	11,547	11,547	11,547	11,547

***p < 0.01, **p < 0.05, *p < 0.1. Standard errors in parentheses are clustered at the community level. Interactive fixed effects are interactive terms for city fixed effects and year fixed effects.

No significant effects were observed on the total caloric intake, as measured by the individuals' dietary energy. Similarly, no significant neighborhood effects were found for carbohydrate intake among the older adults. However, neighborhood CHEI was positively associated with protein intake and lower fat intake. Furthermore, neighborhood CHEI was associated with an improved nutrient structure in older individuals. In particular, older adults in advantaged neighborhoods consumed a greater percentage of calories from protein and a lower percentage of calories from carbohydrates and fat, although the coefficient for carbohydrate was not statistically significant.

### Robustness check

In this section, we perform robustness tests. First, we changed the key variable to another diet quality indicator (DQD indicator) and re-estimated neighborhood effects on eating behaviors of older adults. Similarly, only the coefficients of the neighborhood effects are reported for each regression. Table 5 presents the estimates. Generally, we found a significant and positive effect of the neighborhood's diet quality on older adults' eating behaviors, which is consistent with the previous results in Table 3. Specifically, a higher neighborhood DQD indicated poorer diet quality in the community. Hence, the impact of neighborhood DQD on older adults' daily consumption of vegetables and fruits as well as meat, eggs, and dairy products is negative and statistically significant. In turn, older adults in disadvantaged neighborhoods had a significantly higher cereal intake.

**Table 5:** Estimation of neighborhood effects on dietary behaviors of older adults (fixed effects estimators, DQD indicator).

**Variables**	**Diet quality**	**Food consumption**		
	**DQD**	**Cereals**	**Vegetables and fruits**	**Meat, eggs and dairy products**
	**(1)**	**(2)**	**(3)**	**(4)**
Neighborhood DQD	0.624*** (0.048)	6.391*** (1.226)	−2.299 (1.480)	−1.887*** (0.592)
Control variables	Yes	Yes	Yes	Yes
Individual FE	Yes	Yes	Yes	Yes
Time FE	Yes	Yes	Yes	Yes
Interactive FE	Yes	Yes	Yes	Yes
Observations	11,547	11,547	11,547	11,547

***p < 0.01, **p < 0.05, *p < 0.1. Standard errors in parentheses are clustered at the community level. Interactive fixed effects are interactive terms for city fixed effects and year fixed effects.

Furthermore, the average number of illnesses during the past 4 weeks and awareness of having a healthy diet in the neighborhood were employed as instrumental variables to address potential endogeneity. In the CHNS, respondents were asked whether they had any of these symptoms during the past 4 weeks, including (1) fever, sore throat, cough, (2) diarrhea, (3) stomach ache, (4) asthma, (5) headache/dizziness, (6) joint pain, muscle pain, (7) rash, dermatitis, (8) eye/ear disease, (9) heart disease/chest pain, (10) other infectious diseases, and (11) other non-communicable diseases. We summed the number of these diseases and calculated community-level averages to measure health shocks to neighbors in the past 4 weeks. In addition, respondents were asked about the importance of a healthy diet in their lives. The options included: (1) not important at all (1 point), (2) not very important (2 points), (3) important (3 points), (4) very important (4 points), and (5) the most important (5 points). Similarly, we calculated the mean value of the corresponding indicator at the community level to measure awareness of healthy eating among neighborhood residents. Before presenting the empirical results, we conducted a series of tests on the instrumental variables. Specifically, the instrumental variables for neighborhood effects pass the Sargan–Hansen test for exogeneity. The F-statistic of the first-stage regression of the instruments is well above the rule-of-thumb threshold of 10 for weak instruments (33). Table 6 lists the two-stage least squares estimates. The coefficients of neighborhood CHEI and DQD are both positive, indicating statistically significant neighborhood effects on dietary behaviors among older adults, which is consistent with the fixed effects estimators in Table 3.

**Table 6:** Estimation of neighborhood effects on dietary behaviors of older adults (2SLS estimators).

**Variables**	**CHEI**	**DQD**
	**(1)**	**(2)**
Neighborhood CHEI	0.616*** (0.210)	
Neighborhood DQD		0.570** (0.257)
Control variables	Yes	Yes
Community dummies	Yes	Yes
Time dummies	Yes	Yes
Observations	13,449	13,449

***p < 0.01, **p < 0.05, *p < 0.1. Standard errors in parentheses are clustered at the community level.

### Heterogeneity analysis

Considering the heterogeneity of older adults in China, we further grouped the entire sample based on their gender and place of residence. Table 7 shows that neighborhood effects have a significant positive effect on diet quality and food consumption in both male and female older adults, and the effects do not differ significantly between groups. Hence, both male and female older adults are influenced by neighborhood diet quality. Similarly, there were no significant regional differences, and the diet quality of both urban and rural older adults was positively affected by neighbors' dietary behaviors. However, in terms of food consumption, we observed differences in neighborhood effects between the urban and rural elderly. In particular, the neighborhood effect among the urban elderly showed a significant decrease in cereals and an increase in vegetables and fruits, whereas among the rural elderly, the neighborhood effects were mainly reflected in an increase in meat, egg, and dairy product intake. Thus, we found a positive impact of neighborhood diet quality on dietary behaviors among male and female older adults, as well as among urban and rural older adults.

**Table 7:** Estimation of neighborhood effects on dietary behaviors of older adults (with subsamples, fixed effects estimators).

	**Region**		**Gender**	
	**Urban**	**Rural**	**Male**	**Female**
	**(1)**	**(2)**	**(3)**	**(4)**
CHEI	0.512*** (0.075)	0.686*** (0.057)	0.649*** (0.059)	0.624*** (0.050)
Cereals	−3.237* (1.895)	1.449 (1.237)	−0.463 (1.182)	0.281 (1.198)
Vegetables and fruits	5.874*** (1.872)	1.287 (1.494)	2.192* (1.279)	2.921** (1.365)
Meat, eggs and dairy products	2.590** (1.123)	1.421*** (0.506)	2.125*** (0.627)	1.603*** (0.506)

***p < 0.01, **p < 0.05, *p < 0.1. Standard errors in parentheses are clustered at the community level.

## Discussion

In this study, we examined the relationship between neighborhood diet quality and dietary behaviors in older adults, using four waves of data from the CHNS. In contrast to previous studies that have examined the influence of neighborhood built environments on dietary behaviors, this study considers the influence of social interactions between residents within the same community by investigating neighborhood effects on elderly dietary behaviors.

The results showed that neighborhood diet quality has a significant and positive relationship with dietary behaviors among older adults in China. The neighborhood effects on elderly eating behaviors manifested in improved dietary structure, including decreased consumption of cereals and increased consumption of vegetables and fruits, as well as meat, eggs, and dairy products. In terms of nutrient intake, there was a significant increase in protein intake, and hence, a greater percentage of calories from protein.

To demonstrate the heterogeneity of older adults' dietary behavior, we examined subsamples by gender and place of residence. Overall, the results for the subsample are consistent with the baseline estimates. Diet quality and food consumption were significantly and positively influenced by neighborhood effects for both male and female older people. Older people living in urban areas were influenced by the neighborhood effect to reduce cereal and increase fruit and vegetable intake, whereas older people living in rural areas increased their intake of meat, eggs, and dairy products.

Future policies for improving diet quality should consider neighborhood-level conditions. The neighborhood effect implies that dietary interventions have positive externalities; therefore, policy interventions should not only start from individuals but should also consider the interpersonal impact of policy interventions and make full use of the demonstration effect of group behaviors, especially in rural areas where residents are closely connected and socially interact with one another.

Although this study provides direct evidence of neighborhood effects on dietary behaviors, it does not directly examine the mechanisms by which this link works. Future research could further test and explore social interactions within communities as a potential channel to explain the importance of the social environment on individual health and dietary behaviors.

## Data availability statement

Publicly available datasets were analyzed in this study. This data can be found here: China Health and Nutrition Survey https://www.cpc.unc.edu/projects/china.

## Author contributions

CL: conceptualization, methodology, formal analysis, writing—original draft, writing—review and editing, and funding acquisition. HY: writing—original draft and writing—review and editing. All authors read and approved the final manuscript.

## Funding

This work was financially supported by the National Natural Science Foundation of China (Grant: 72003091), Project of Philosophy and Social Science in Colleges and Universities of Jiangsu Province (Grant: 2022SJYB0158), and the Special Project of Ecological Civilization Construction Research in Humanities and Social Sciences of Nanjing Forestry University (Grant: A2021YB13).

## Conflict of interest

The authors declare that the research was conducted in the absence of any commercial or financial relationships that could be construed as a potential conflict of interest.

## Publisher's note

All claims expressed in this article are solely those of the authors and do not necessarily represent those of their affiliated organizations, or those of the publisher, the editors and the reviewers. Any product that may be evaluated in this article, or claim that may be made by its manufacturer, is not guaranteed or endorsed by the publisher.

## Footnotes

^1^The latest wave is not included in this study because data from the dietary questionnaire have not been published in 2015.

^2^Healthy Eating Index (HEI) has been widely used in previous studies for assessing diet quality for Americans.

^3^Cooking oils and alcohol are measured in grams per 1,000 calories (g/1,000 kcal), whereas sodium is measured in milligrams per 1,000 calories (mg/1,000 kcal).

^4^Owing to data availability in China Food Composition, added sugars is not included in this study. Following Liu et al. (27), we reassigned Dairy with 10 points (instead of 5 points) to ensure that the maximum total score of CHEI is 100 points.

^5^Following Tian and Yu (30), older adults with an average daily calorie intake below 520 kcal (energy requirement for a newborn baby) and above 4,046 kcal were dropped in this study (exceeding three standard deviations in the sample).
